# Fluoride-doped amorphous calcium phosphate nanoparticles as a promising biomimetic material for dental remineralization

**DOI:** 10.1038/s41598-018-35258-x

**Published:** 2018-11-19

**Authors:** Michele Iafisco, Lorenzo Degli Esposti, Gloria Belén Ramírez-Rodríguez, Francesca Carella, Jaime Gómez-Morales, Andrei Cristian Ionescu, Eugenio Brambilla, Anna Tampieri, José Manuel Delgado-López

**Affiliations:** 1 Institute of Science and Technology for Ceramics (ISTEC), National Research Council (CNR), Via Granarolo 64, 48018 Faenza, Italy; 2 Department of Chemistry, Life Sciences and Environmental Sustainability, University of Parma, Parco Area delle Scienze 17/a, 43124 Parma, Italy; 3 Departamento de Química Inorgánica, Universidad de Granada, Av. Fuente Nueva, s/n, 18071 Granada, Spain; 4 Laboratorio de Estudios Cristalográficos, Instituto Andaluz de Ciencias de la Tierra, IACT (CSIC-UGR), Av. Las Palmeras 4, 18100 Armilla, Spain; 5 Oral Microbiology Laboratory, Galeazzi Orthopedic Institute, Department of Biomedical, Surgical and Dental sciences, University of Milan, Via Pascal, 36, 20133 Milan, Italy

## Abstract

Demineralization of dental hard tissue is a widespread problem and the main responsible for dental caries and dentin hypersensitivity. The most promising strategies to induce the precipitation of new mineral phase are the application of materials releasing gradually Ca^2+^ and PO_4_^3−^ ions or mimicking the mineral phase of the host tissue. However, the design of formulations covering both processes is so far a challenge in preventive dentistry. In this work, we have synthesized innovative biomimetic amorphous calcium phosphate (ACP), which has been, for the first time, doped with fluoride ions (FACP) to obtain materials with enhanced anti-caries and remineralizing properties. Significantly, the doping with fluoride (F) did not vary the physico-chemical features of ACP but resulted in a faster conversion to the crystalline apatite phase in water, as observed by *in-situ* time-dependent Raman experiments. The efficacy of the as synthesized ACP and FACP samples to occlude dentinal tubules and induce enamel remineralization has been tested *in vitro* in human molar teeth. The samples showed good ability to partially occlude the tubules of acid-etched dentin and to restore demineralized enamel into its native structure. Results demonstrate that ACP and FACP are promising biomimetic materials in preventive dentistry to hinder demineralization of dental hard tissues.

## Introduction

Despite the remarkable advances in oral care technology, demineralization of dental hard tissues (enamel and dentin) is still a growing issue and the main responsible for dental caries and dentin hypersensitivity^1^. It is caused by a low pH environment which, in turn, is the consequence of the intake of acidic food or drinks, the presence of gastroesophageal reflux disease (GERD) and/or the acidogenic activity of a pathogenic oral biofilm^2^. When the pH of the saliva drops below 5.5, hydroxyapatite (HA), representing 95 wt% and 75 wt% of enamel and dentinal tissue respectively, starts to dissolve^3,4^.

Demineralization is a reversible process if the damaged tissues are exposed to oral environment that favors remineralization. For example, the enamel cavities caused by demineralization processes are naturally remineralized by the epitaxial growth of residual crystals acting as nucleation sites, while saliva provides a supersaturated Ca^2+^ and PO_4_^3−^ ions environment respect to HA^1,5^. However, remineralization of enamel by saliva is seldom completely achieved, especially when there is an imbalance in duration and extent of demineralization/remineralization phases^1^.

Therefore, it is required the use of an external source of Ca^2+^ and PO_4_^3−^ ions to increase the HA supersaturation to efficiently prevent demineralization and boost remineralization^5^. It can be also achieved with the use of materials chemically similar to those present in the dental tissue which could promote the natural repair of the damaged tissues^2^.

Various forms of biomimetic calcium phosphates (CaPs), mimicking the composition and structure of the mineral phase of bone and teeth (the so called “biomimetic”), such as: HA, fluoro-hydroxyapatite (FHA), and amorphous calcium phosphates (ACP) have been proposed for dental hard tissue remineralization^3–7^. These agents can be added to restorative materials or directly applied onto the tooth surface^5^. It has been demonstrated that nanostructured CaPs are more efficient for remineralization therapies in comparison to their macro-sized counterparts due to their biomimetism, higher surface area and reactivity as well as better ability to adhere and penetrate into the enamel and dentinal lesions^8,9^.

Among CaPs, ACP nanoparticles are particularly appealing in dentistry due to their ability to release a significant amount of Ca^2+^ and PO_4_^3−^ ions, as compared with other crystalline CaP phases. ACP is a mineral phase with a short-range order, and its basic structural unit, as proposed by Betts and Posner, is a roughly spherical cluster of ions having an average diameter of 0.95 nm consistent with the chemical composition Ca_9_(PO_4_)_6_^10,11^. It is an unstable material that rapidly transforms into the more thermodynamically stable CaP phases (i.e., HA and octacalcium phosphate (OCP)) in solution or in dry state by reacting with atmospheric water^12^. ACP is a precursor (transient) phase of biogenic HA of bone and tooth^6,9,13,14^. Indeed, it was demonstrated by Robinson *et al*. that the formation of enamel occurs through the deposition of spherical ACP nanoparticles into chains, being subsequently transformed into HA^15^.

Thanks to its excellent bioactivity, high cell adhesion, tailorable biodegradation and good osteoconductivity, ACP is not only studied for dental applications but it is currently employed to manufacture several biomaterials for bone repair^16,17^. It is used, for example, in the preparation of coatings on metallic prostheses, self-setting injectable cements and hybrid composites when mixed with polymers^14,16,17^.

Since ACP can readily convert to crystalline phases, its use and handling is difficult. Therefore, several additives and ions were studied to stabilize ACP including: casein phosphopeptides (CPP)^18^, carboxymethyl chitosan (CMC)^19,20^, polyethylene glycol^21^, polyaspartic acid^22^, adenosine triphosphate (ATP)^23^, magnesium ions^24^, and poly(ethylene glycol)-block-polylactide (PEG-PLA)^25^. To our knowledge, most of these materials are not available on the market due to difficulties in the scale up process, costs and concerns on the biocompatibility of the stabilizing agents. Only CPP-stabilized ACP (CPP-ACP) is actually marketed for enamel remineralization in a formulation that is directly applied on tooth surface (i.e. tooth mousse), having a good level of literature evidence for this use (systematic reviews)^2,26^. Additionally, CPP-ACP is employed as an effective dentin desensitizer, where its action is to fill and occlude dentinal tubules^27,28^. The hypersensitivity deriving from open dentinal tubules is indeed one of the worst consequences of dentinal demineralization^28^. However, CPP is a milk-derived protein and it cannot be used by patients having intolerance to milk^29^.

Fluoride is the most employed prophylactic agent to reduce and prevent enamel demineralization, remaining so far as the most effective agent for caries prevention^30^. It is considered to act by two different mechanisms^31,32^: i) replacing of the hydroxyl groups of the new formed HA, resulting in fluorapatite [FHA, Ca_5_(PO_4_)_3_F], which is less soluble and thus more resistant to low pH values than HA^32^; ii) inhibiting the metabolic and physiological pathways of microorganisms in the cariogenic biofilm that produce organic acids to demineralize dental tissue^32^. In addition to that, an inverse relationship can be found between the concentration of Ca^2+^ in oral biofilms and dental caries risk, therefore several Ca^2+^-containing compounds were introduced in oral care products and dental materials to promote remineralization^33^. These compounds have exhibited a relevant effect on oral biofilms, especially depending on the composition and surface characteristics of the material itself ^9,34^.

The aim of this work is the preparation of new forms of biomimetic ACP nanoparticles as a promising nanomaterial for dental remineralization. The biomimetic nanoparticles can play a dual role in preventive dentistry: i) occlusion of the dentinal tubules due to their nano-dimensions and ii) delivery of Ca^2+^, PO_4_^3−^ directly on hard tissue surfaces, generating a local supersaturation that triggers the remineralization of dental hard tissues. Our bioinspired synthesis uses citrate ions, which have been shown to play an important role in stabilizing the precursor ACP phase^35–39^. Recent studies have evidenced the relatively large amount of citrate in bone, where it accounts for ~5.5% wt of the total organic component (ca. 2% of the total amount), and found to be strongly bound to apatite platelets, controlling their size and morphology^40,41^. Although in a less extent, it has been also found in dentin and enamel^42,43^.

We have additionally explored the possibility of incorporating fluoride ions in the ACP nanoparticles (FACP) without affecting their amorphous nature in order to generate materials with potentially enhanced anti-caries and remineralizing properties. Indeed, the topical delivery of fluoride mediated by ACP nanoparticles directly in the dental cavities/lesions, could enhance its efficacy and reduce side effects. Actually, the overconsumption of fluoride through fluoridated water/foods and supplements may lead to dental or skeletal fluorosis^44^.

We have studied the time-depended release of Ca and F in artificial saliva and monitored the (F)ACP-to-(F)HA conversion by time-dependent *in-situ* Raman spectroscopy. Results confirm that the ACP and FACP nanoparticles are able to efficiently occlude uncovered dentinal tubules and remineralize damage on human molar teeth. This implies that ACP and FACP are suitable for the remineralization of dental hard tissues since they were able to restore enamel in its native structure.

## Results and Discussion

### Morphological, structural, and compositional characterization of ACP

**Figure 1:**
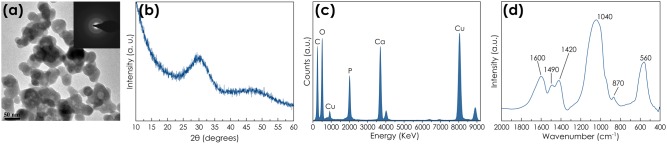
Morphological, structural and compositional characterization of ACP-4 nanoparticles. (**a**) TEM micrograph (inset: SAED pattern), (**b**) XRD pattern, (**c**) EDS spectrum and (**d**) FTIR spectrum.

TEM micrograph of ACP-4 (Fig. 1(a)) reveals the formation of round shaped nanoparticles, with sizes ranging between 20 and 50 nm and amorphous in nature as indicated by the diffuse broad rings of the SAED patterns (inset in Fig. 1(a)) and the broad band at about 30° (2θ) in the XRD pattern (Fig. 1(b)). This confirms the lack of long-range periodicity and thus excludes the presence of HA or others CaP crystalline phases.

In addition, EDS spectrum (Fig. 1(c)) corroborates that they are composed of calcium and phosphate. FT-IR spectrum (Fig. 1(d)) displays broad bands characteristic of ACP. In particular, the absorption bands at ca. 560 and 1050 cm^−1^ are associated to the bending and stretching modes of phosphate groups, respectively; those at ca. 870 cm^−1^ and in the range 1400–1500 cm^−1^ are attributed to the carbonate ions, while the band at ca. 1600 cm^−1^ can be assigned to the adsorbed water and to the stretching of -COO^−^ of citrate^39^.

The Ca/P molar ratio of ACP-4 (i.e., 1.70) is higher than 1.50, which is the one of the highest value found for ACP obtained in alkaline media^16^. However Ca/P values similar to that of ACP-4 were previously reported for ACP samples containing foreign ions like citrate and carbonate, as in the case of the present study^16^. TGA curve of ACP-4 (Fig. S2, Supplementary information (SI)) mainly exhibits four weight losses, that can be attributed to adsorbed water (from room temperature to 150 °C), structural water (from 150 to 350 °C), citrate (from 350 to 700 °C) and carbonate (from 700 to 1000 °C), in agreement with the TGA curve of citrate-HA nanoparticles previously reported^35^. According to these losses the content of citrate and carbonate were estimated (Table 1). The value of SSA_BET_ was in line with the nano-dimensions detected by TEM but remarkably higher than that of other type of ACP nanoparticles previously described^45,46^.

**Table 1:** Chemical composition and specific surface area (SSA_BET_) of the powder samples.

Sample	Ca (wt%)^a^	P (wt%)^a^	Ca/P (mol)^a^	F (wt%)^b^	Citrate (wt%)^c^	Carbonate (wt%)^c^	SSA_BET_ (m^2^ g^−1^)^d^
ACP-4	29.9 ± 0.7	13.6 ± 0.3	1.70 ± 0.02	—	1.9 ± 0.2	3.7 ± 0.4	200 ± 20
FACP-l4	31.6 ± 0.7	14.4 ± 0.3	1.70 ± 0.01	0.10 ± 0.01	2.2 ± 0.2	3.2 ± 0.3	255 ± 26
FACP-h4	31.4 ± 0.4	13.6 ± 0.2	1.78 ± 0.01	1.00 ± 0.10	1.5 ± 0.2	3.8 ± 0.4	213 ± 21
ACP-2	29.1 ± 1.0	13.2 ± 0.3	1.70 ± 0.02	—	2.2 ± 0.2	3.8 ± 0.4	287 ± 29
FACP-h2	32.1 ± 0.5	13.1 ± 0.2	1.89 ± 0.01	1.10 ± 0.10	2.0 ± 0.2	3.4 ± 0.3	328 ± 33
ACP-1	28.0 ± 0.6	12.7 ± 0.2	1.70 ± 0.04	—	1.8 ± 0.2	3.2 ± 0.3	309 ± 31
FACP-h1	31.9 ± 0.8	13.1 ± 0.3	1.88 ± 0.01	1.30 ± 0.10	2.4 ± 0.2	3.1 ± 0.3	293 ± 29

^a^Quantified by ICP-OES; ^b^Quantified with a fluoride ion selective electrode; ^c^Quantified by TGA; ^d^Calculated from BET adsorption.

The stability of ACP-4 powder stored at room temperature up to one year has been evaluated analyzing its structure collecting the XRD pattern (Fig. S3, SI). Interestingly, the XRD pattern remained unchanged, establishing that the amorphous nature of ACP-4 is preserved during this period of time. This long-term stability in dry state is ascribed to the presence of citrate ions, which has been previously proposed to play an important role in stabilizing the amorphous precursor^38,47,48^ as well as in controlling the thickening of bone apatite platelets by specific adsorption^40^. As discussed before, the use of citrate, naturally present in bone^40,49^, enamel and dentine^42,49^, as stabilizing agent enhances the “*biomimetism*” of our ACP nanoparticles.

### Morphological, structural and compositional characterization of FACP

It is well reported that the presence of carbonate and/or magnesium favors the formation of ACPs and slows down their further transformation to crystalline phase^16^. However, fluoride has the opposite effect, favoring the formation of the more thermodynamically stable FHA^50^. Indeed, to the best of our knowledge, the synthesis of stable fluoride-doped ACP has not been reported so far. In a first step, maintaining the experimental conditions of ACP-4, we added two different concentrations of NaF in the phosphate-containing solution with the aim of doping ACP-4 with increasing F contents.

**Figure 2:**
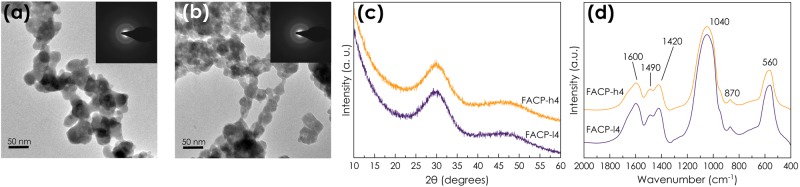
Morphological, structural and compositional characterization of FACP nanoparticles. TEM micrograph of (**a**) FACP-h4 and (**b**) FACP-l4 nanoparticles (insets: SAED patterns); (**c**) XRD patterns and (**d**) FTIR spectra of FACP-l4 (magenta) and FACP-h4 (orange) nanoparticles.

TEM images of FACP-h4 (Fig. 2(a)) and FACP-l4 (Fig. 2(b)) also show round-shaped nanoparticles, very similar in size and morphology to ACP-4. The presence of broad reflections in the SAED (insets of Fig. 2(a,b)) and XRD patterns collected on these particles (Fig. 2(c)) confirms their amorphous nature. FT-IR spectra of FACP-l4 and FACP-h4 also display broad, unresolved bands similar to those observed in the FT-IR spectrum of ACP-4 (Fig. 2(d)). Therefore, we can conclude that adding F^−^ ions did not cause the precipitation of fluoride salts or other crystalline CaP phases as previously reported in the absence of citrate^16,50^.

The chemical analysis of FACP-l4 and FACP-h4 confirms the incorporation of F into the ACP nanoparticles. The amount of fluorine linearly increases with the initial NaF concentration, being ten times higher in FACP-h4 with respect to FACP-l4 (Table 1). Fluorine incorporation results in an increase of the calcium content, and thus Ca/P ratio (Table 1), which is likely a consequence of the neutralization of the negative density charge of fluoride ions. Indeed, the increase in Ca/P ratio was observed when a relatively high content of F^−^ is incorporated (FACP-h4), but not when the F content is low (FACP-l4) (Table 1). The amount of citrate and carbonate calculated by TGA as well as the SSA_BET_ were not affected by the incorporation of fluorine (Table 1). It is also worth mentioning that the XRD patterns of dry fluoride-doped ACP remained unchanged during, at least, one year (Fig. S3, SI). This observation confirms their high stability in dry state at room temperature.

We have also explored the impact of the Cit/Ca ratio in the physico-chemical features of the F-doped and un-doped ACP. We found out that the morphological, structural and compositional features as well as long-term stability in dry state are practically independent of the initial Cit/Ca ratio (Table 1 and Fig. S3–S5, SI). The specific surface area (SSA_BET_) is the only value clearly affected when varying the Cit/Ca ratio, i.e., SSA_BET_ increases when decreasing Cit/Ca ratio. SSA_BET_, value of ACP-1 (Cit/Ca = 1, Table 1) is notably high (328 m^2^ g^−1^). To the best of our knowledge, such high value has never been reported for CaP based materials. Since ACP-1/2/4 samples are similar in size and morphology, this increase in SSA_BET_ could be due to differences in the porous structure. MIP analysis on ACP-4 and ACP-1 powders (Fig. S6, SI) revealed that the latter has a higher porosity than ACP-4 (92.52% *vs* 84.19%.). The pore size distribution shows that both samples possess micro- and nanopores of about 35 µm and 55 nm in diameter for ACP-4 and of about 20 µm and 20 nm for ACP-1. Moreover, the nanopores of ACP-1 constitute a higher percentage of the total porosity compared to ACP-4. These differences in porosity could explain the increase in SSA observed for ACP-1.

### (F)ACP to (F)HA conversion kinetics and ion releases in acidified artificial saliva

The application of ACP for teeth remineralization is based on two principles: (i) gradual release of Ca^2+^ and PO_4_^3−^ ions generating a local supersaturation that triggers the remineralization of hard tissues and (ii) attachment to the surface of the hard tissues, being then transformed to HA. Therefore, we have evaluated *in vitro* the capabilities of the ACP and FACP samples in inducing both effects.

**Figure 3:**
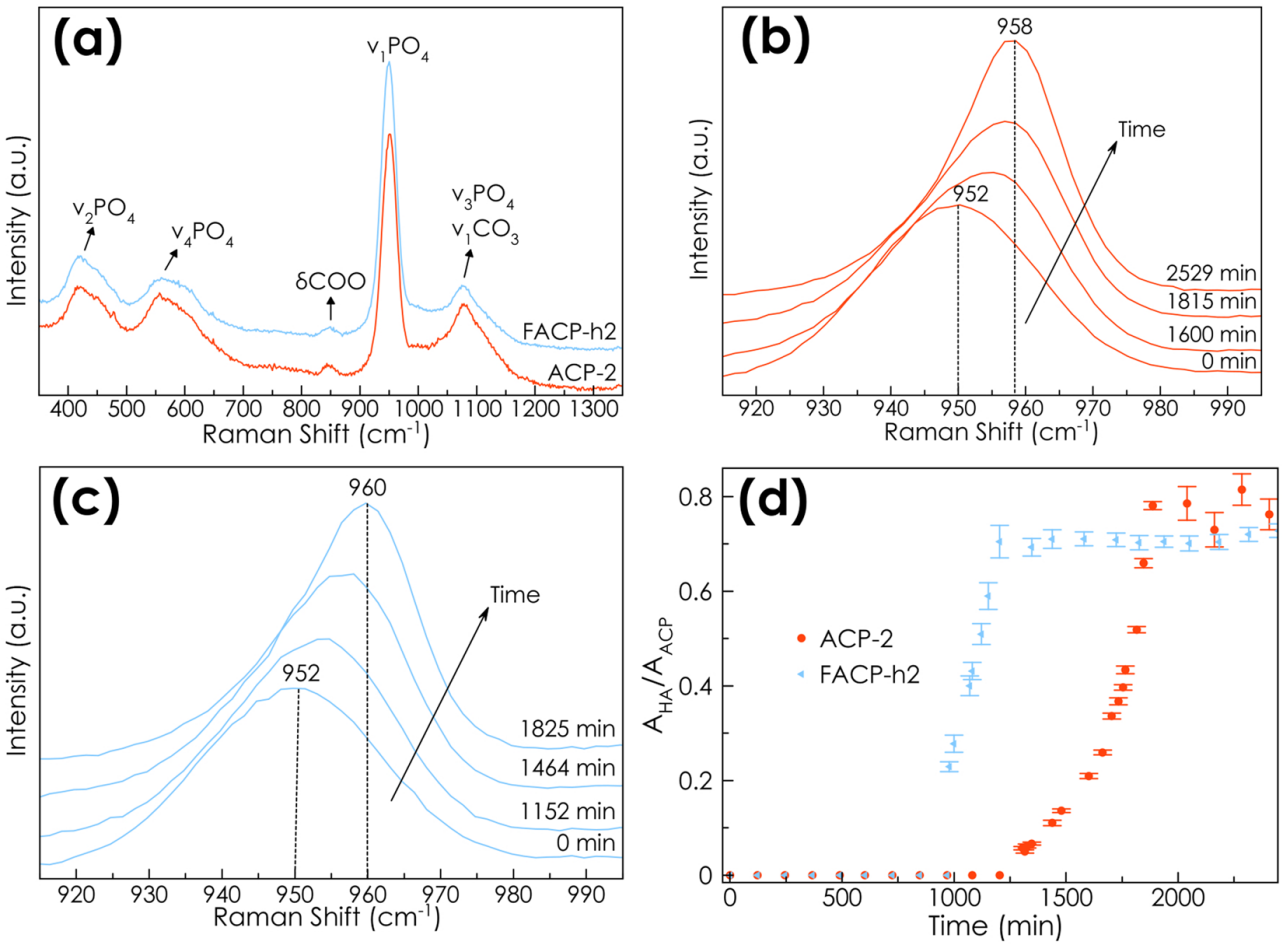
Monitoring the (F)ACP-to-(F)HA conversion by *in-situ* Raman micro-spectroscopy. (**a**) Raman spectra of ACP-2 (red) and FACP-h2 (blue). *In situ* time-dependent Raman spectra (ν_1_PO_4_ vibrations) collected during the transformation of (**b**) ACP-2 and (**c**) FACP-h2 to HA and FHA, respectively, in water. (**d**) Time-dependent intensity ratio of the ν_1_PO4 peak of ACP-2/ FACP-h2 (952 cm^−1^) and HA/FHA (959 cm^−1^).

Raman spectra of dry ACP-2 and FACP-h2 are shown in Fig. 3(a). They exhibit the characteristics Raman peaks of phosphate vibrations of ACP along with the υ_1_CO (carbonate) and δCOO (citrate) vibrational modes^38^. Concretely, the main peak, due to the ν_1_PO_4_ vibration, appears at *ca*. 952 cm^−1^, in agreement with the amorphous nature of the nanoparticles. On the contrary, this peak appears at ca. 958 cm^−1^ for nanocrystalline apatite^38^ and 962 cm^−1^ for nanocrystalline fluoroapatite^51^. Therefore, the blue-shift from 952 to 958/960 can be used to monitor the conversion from (F)ACP to (F)HA in aqueous media^38^. Time-dependent *in-situ* Raman spectra (ν_1_ PO_4_ region) of ACP-2 and FACP-2 in water are shown in Fig. 3(b,c), respectively. A peak upshift from 950 cm^−1^ to ca. 958 cm^−1^ (HA) and 960 cm^−1^ (FHA) is clearly visible, indicating the existence of a gradual transformation from (F)ACP to (F)HA, respectively. The normalized ratio of the corresponding ν_1_PO_4_ Raman band of (F)HA and ACP (i.e., A_960_/A_950_) was used to study the extent of the conversion (Fig. 3(c)). As expected, we found that F-doing resulted in a much faster (F)ACP-(F)HA conversion. The FACP-h2 sample was transformed into FHA after 18 hours, while the ACP-2 sample required around 32 hours to be completely transformed into HA (Fig. 3(d)). The conversion kinetic of ACP-4 was previously reported^38^. The comparison of ACP-4 and ACP-2 turns out that increasing the citrate concentration ends up in a slower conversion, since ACP-4 is transformed after 54 hours.

**Figure 4:**
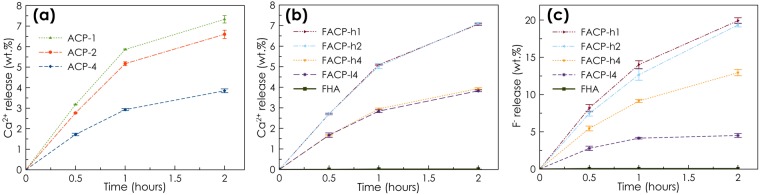
*In vitro* release of Ca and F in acidic artificial saliva. Cumulative Ca^2+^ ions release from (**a**) ACP samples and (**b**) FACP samples (B). (**c**) Cumulative F^−^ ions release from FACP samples. The cumulative release of Ca and F from crystalline FHA is also shown in (**b**) and (**c**), respectively.

The ions (Ca^2+^ and F^−^) release has been evaluated *in vitro* in acidified artificial saliva, an organic-free solution that mimics the composition of human saliva after eating (Fig. 4). (F)ACP samples show a gradual and progressive release of Ca^2+^ and F^−^ ions. Comparatively, the Ca^2+^ and F^−^ release from a sample of crystalline FHA – a material that is commonly used as tooth treatment products – is negligible under the same conditions, which confirms the high efficiency of (F)ACP for the gradual release of Ca^2+^ and F^−^ ions. The samples prepared with a lower Cit/Ca ratio provided higher ion release rates probably due to their higher SSA_BET_. In fact, all the samples with similar surface area (*i.e*. ACP-2, ACP-1, FACP-h2 and FACP-h1) present comparable Ca^2+^ and F^−^ release kinetic.

### *In vitro* test of remineralization and dentinal tubules occlusion

We have tested *in vitro* the effect of ACP-4/1 and FACP-h4/1 samples as remineralization materials, evaluating their potential application for dentinal tubules occlusion and enamel remineralization. Samples of human enamel and dentin were etched with H_3_PO_4_ simulating an early enamel/dentine lesion, and subsequently treated with concentrated slurries of ACP and FACP for 24 h.

**Figure 5:**
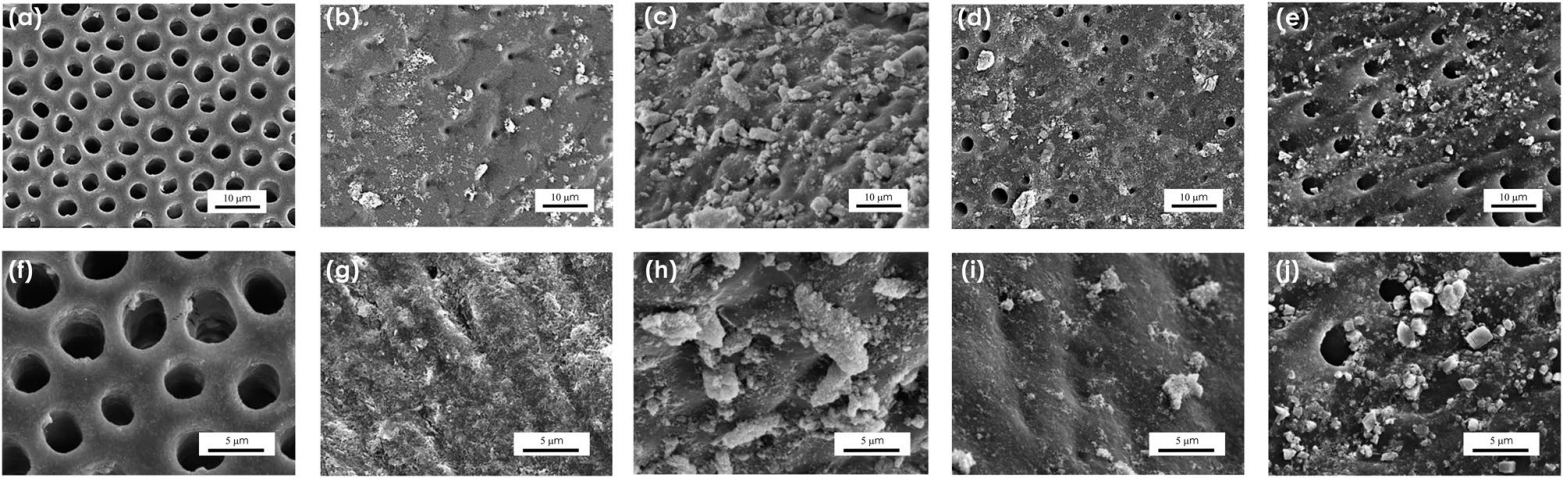
SEM observations of dentin remineralization. SEM micrographs of (**a**,**f**) demineralized dentin and demineralized dentin treated with (**b**,**g**) ACP-4, (**c**,**h**) FACP-h4, (**d**,**i**) ACP-1, and (**e**,**j**) FACP-h1 at two different magnifications.

ACP and FACP samples showed good behavior on occluding dentinal tubules, as seen in Fig. 5. Specifically, ACP samples displayed a better activity providing a higher degree of occlusion than FACP (Fig. 5(b–e)). According to *in-situ* Raman observations, ACP is still stable after 24 hours in water, while FACP has been completely converted into FHA. The higher stability of ACP in water could explain its better activity in occluding the tubules. The faster transformation occurring in the FACP sample could marginally hinder the penetration in the tubules at 24 h. This is especially evident in the dentin treated with FACP-h1 (Fig. 5(e,j) and Fig. S7 of SI), which showed the poorest occluding effect and the highest presence of crystalline micro-particles. It must be highlighted, nevertheless, that all samples showed a relevant occluding effect.

**Figure 6:**
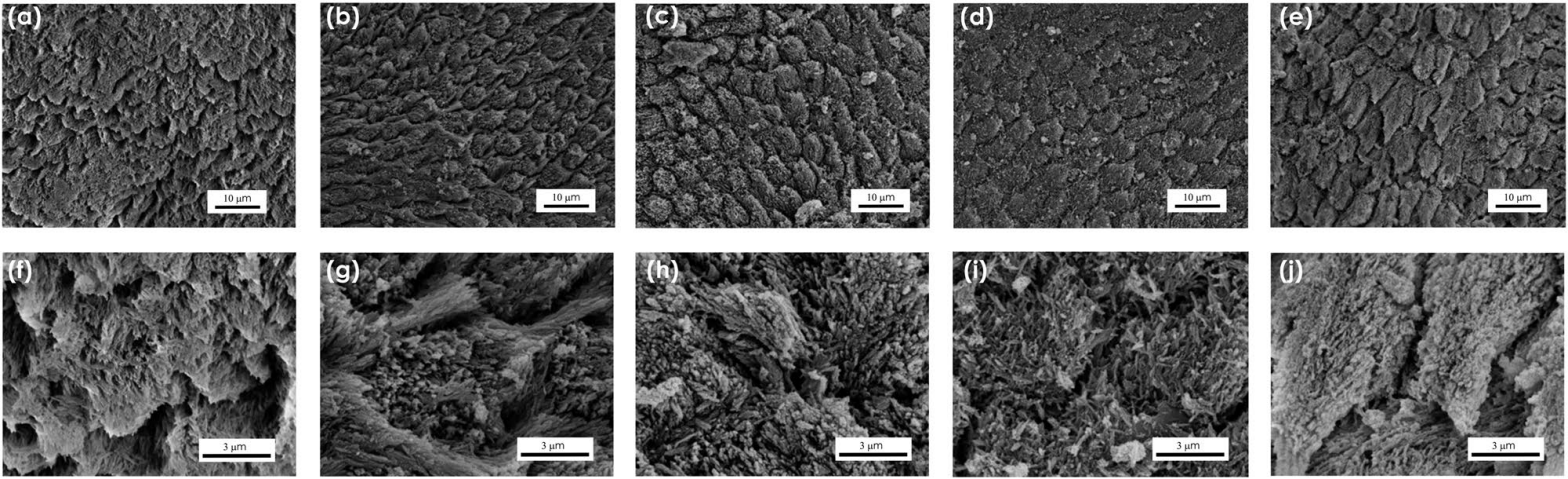
SEM observations of enamel mineralization. SEM micrographs of (**a**,**f**) demineralized enamel and demineralized enamel treated with (**b**,**g**) ACP-4, (**c**,**h**) FACP-h4, (**d**,**i**) ACP-1, and (**e**,**j**) FACP-h1 at two different magnifications.

Regarding the enamel remineralization effect, some nano- or microcrystals of HA growing onto the ordered microcrystals of enamel prisms are clearly visible (Fig. 6). The newly deposited crystals are less ordered than the native ones, but it is remarkable that they form a layer of enamel-like crystals, mimicking those of the biological sample which acts as substrate. In this sense, the remaining microstructures of the dental tissue may have acted as a remineralization guide possibly meaning that remineralization of damaged tissues may achieve better performances if residual microstructures of the tissue are not altered. This implies that ACP and FACP are suitable for enamel remineralization, since they were able to restore enamel in its native structure. This is a notable advantage over other materials that only act as fillers of demineralized regions without any regeneration effect^13^. It is worth mentioning that the interprismatic regions seem to be restored and less hollowed with the ACP samples (Fig. 6(b,d) and Fig. S8(b,d) in SI); probably ACP displays a remineralization and regenerative effect similar to what has been observed at lower magnification on dentine (Fig. S7, SI). This effect is particularly interesting since the interprismatic regions, being less crystalline, are the ones more subjected to acidic erosion^52,53^. On the other hand, FACP samples showed an enhanced remineralization effect on prismatic enamel (Fig. 6(c,e) and Fig. S8(c,e) in SI). It may be argued that the quickly formed FHA crystals tend to adhere and grow on highly crystalline prismatic regions. Further studies may investigate the effect of this remineralizing technology when associated with a scaffold technology that may mediate ions release over an extended amount of time. Moreover future analysis on the effect of F ions delivered by ACP compared to free F ions on the resistance of enamel to low pH will be carried out.

## Conclusions

Innovative biomimetic ACP and FACP nanoparticles (with tunable F^−^ contents), acting as promising dentin desensitizer and remineralizing agent in preventive dentistry have been produced using a straightforward and scalable synthetic route. The presence of citrate makes them stable in dry state at least up to one year. Reducing the Cit/Ca molar ratio of the reactants from 4 to 2 (or 1), the specific surface area significantly increases, leading to particles with faster Ca^2+^ and F^−^ release kinetics and conversion into HA. Significantly, the presence of fluoride did not induce any significant change in the chemical-physical features of ACP, apart from the more rapid conversion in the crystalline phase when immersed in aqueous solution. As proof of concepts, the efficacy of the ACP and FACP samples for enamel remineralization and occlusion of dentinal tubules has been tested *in vitro*. All the samples showed good ability to partly occlude the tubules after 24 h of contact with acid-etched dentin samples. ACP samples displayed a faster activity compared to FACP due to their slower conversion kinetic to HA revealing an optimal agent in the case of severe dentinal hypersensitivity. We have demonstrated that ACP and FACP are able to restore enamel in its native structure. In particular, FACP samples showed a faster remineralization effect compared to ACP on prismatic enamel thus being suitable for patients exhibiting severe enamel demineralization. Overall, our results show that the use of fluoride and citrate allow to tune specific physico-chemical properties of ACP and FACP, devoted to specific needs of the patient. These data are encouraging and open the doors to future clinical studies that will allow to better validate the therapeutic value of ACP and FACP.

## Methods

### Materials

Calcium chloride dihydrate (CaCl_2_∙2H_2_O, ≥99.0% pure), sodium citrate tribasic dihydrate (Na_3_(C_6_H_5_O_7_)∙2H_2_O, ≥99.0% pure (Na_3_(Cit)), sodium phosphate dibasic dihydrate (Na_2_HPO_4_∙2H_2_O, ≥99.0% pure), sodium carbonate monohydrate (Na_2_CO_3_∙2H_2_O, ≥99.0% pure), sodium fluoride (NaF, ≥99.0% pure), potassium chloride (KCl ≥ 99.5% pure), potassium thiocyanate (KSCN ≥ 98.0% pure), sodium carbonate monobasic (NaHCO_3_, ≥99.7% pure), orthophosphoric acid (H_3_PO_4_ 85 wt% in H_2_O) and lactic acid (C_3_H_6_O_3_ ≥ 90.0% pure) were purchased from Sigma Aldrich (St. Luis, MO, USA). Fluoro-hydroxyapatite (FHA) was purchased from Kalichem S.r.l. (Rezzato, BS, Italy). All the solutions were prepared with ultrapure water (0.22 µS, 25 °C, MilliQ^©^, Millipore).

### Sample preparation

Dry powder ACP-4 was synthesized by mixing two solutions (1:1 v/v, 200 ml total) at room temperature of (i) 100 mM CaCl_2_ + 400 mM Na_3_(Cit) and (ii) 120 mM Na_2_HPO_4_ + 200 mM Na_2_CO_3_. The pH was adjusted to 8.5 with HCl solution. When the mixture became milky (approximately 30 seconds after mixing), the particles were repeatedly washed with ultrapure water by centrifugation (5000 rpm, 15 min, 4 °C) and then freeze-dried overnight at −50 °C under a vacuum of 3 mbar. FACP samples were prepared similarly to ACP, but adding 5 or 50 mM NaF to the solution (ii) (referred to as FACP-l4 and FACP-h4, respectively). Samples of ACP and FACP – doped with the highest nominal content of F^−^ were also prepared decreasing the initial molar Cit/Ca ratio to 2 and 1 (referred to as ACP-2, FACP-h2 and ACP-1, FACP-h1, respectively). Codes of the samples and the corresponding concentrations of the chemical reactants used for their preparation are reported in Table S1 of SI.

### Chemical, morphological and structural characterization

X-ray diffraction (XRD) patterns of the samples were recorded on a D8 Advance diffractometer (Bruker, Karlsruhe, Germany) equipped with a Lynx-eye position sensitive detector using Cu Kα radiation (λ = 1.54178 Å) generated at 40 kV and 40 mA. Spectra were recorded in the 2θ range from 10 to 60° with a step size (2θ) of 0.021 and a counting time of 0.5 s.

Fourier transform infrared (FT-IR) spectroscopy analyses were carried out on a Nicolet 5700 spectrometer (Thermo Fisher Scientific Inc., Waltham, MA, USA) with a resolution of 2 cm^−1^ by accumulation of 64 scans covering the 4000 to 400 cm^−1^ range, in transmission mode using KBr pellets.

Transmission electron microscopy (TEM) evaluation was performed with a Tecnai F20 microscope (Fei Corp., Hillsboro, OR, USA) operating at 120 kV. The powder samples were ultrasonically dispersed in ultrapure water and then a few droplets of the slurry were deposited on 200 mesh copper TEM grids covered with thin amorphous carbon films and incubated for several minutes. After that, the excess of water was manually blotted.

Time-dependent Raman spectra were collected on ACP and FACP samples in contact with water to monitor the ACP-to-HA conversion. ACP or FACP suspensions were drop-cast between a glass slide and glass slip that were carefully sealed with vacuum grease in order to prevent evaporation during the acquisition. Subsequently, Raman spectra were sequentially collected under back scattering geometry with a LabRAM-HR spectrometer (Jobin-Yvon, Horiba, Japan). The excitation line was provided by a diode laser emitting at a wavelength of 532 nm and a Peltier cooled charge-couple device (CCD) (1064 × 256 pixels) was used as detector. A diffraction grating of 600 T grooves/mm was used. The final spectrum resulted by the average of 3 acquisitions (acquisition time = 250 s). Curve fitting, using gaussian functions, and quantification of integral areas were done using MagicPlotPro (2.5.1) software. Quantification of Ca and P content was carried out by inductively coupled plasma atomic emission (ICP-OES) spectrometer (Agilent Technologies 5100 ICP-OES, Santa Clara, CA, USA) while F was quantified with a fluoride ion selective electrode (Intellical™ ISEF121, Hach Lange, Loveland, CO, USA). In both cases, samples were prepared dissolving an aliquot of powder in a 1 wt% HNO_3_ solution.

Scanning Electron Microscopy (SEM) imaging and Energy-dispersive X-ray spectroscopy (EDS) was performed with a field-emission microscope (FESEM, mod. ΣIGMA, ZEISS NTS Gmbh, Oberkochen, Germany) coupled to an energy-dispersive X-ray microanalyzer (mod. INCA Energy 300, Oxford Instruments, Abingdon- on-Thames, UK). Samples were fixed on aluminum stubs using a carbon tape.

Thermogravimetry analyses (TGA) were performed using a STA 449 Jupiter (Netzsch GmbH, Selb, Germany) apparatus. About 10 mg of sample was weighted in a platinum crucible and heated from room temperature to 1200 °C under air flow with a heating rate of 10 °C/min.

Brunauer-Emmett-Teller (BET) N_2_ gas adsorption method was employed to measure the specific surface area (SSA) of powdered samples using a Sorpty 1750 instrument (Carlo Erba, Milan Italy).

The pore size distribution in the range 0.0070–100 μm was analyzed by mercury intrusion porosimetry (MIP) using Pascal 140 and 240 porosimeters (Thermo Fisher Scientific Inc., Waltham, MA, USA) with a surface tension of 0.48 N/m and a contact angle of 140°. The measurements were performed on the powder samples, with an experimental error of 4% due to the accuracy of the instrument.

### Ion release in acidic artificial saliva

A total of 200 mg of ACP, FACP or FHA powders were dispersed into 10 mL of artificial saliva prepared as modified Tani-Zucchi solution^54,55^ containing KCl 20 mM, KSCN 5.3 mM, Na_2_HPO_4_ 1.4 mM, NaHCO_3_ 15 mM, and lactic acid 10 mM. The suspension was maintained at 37 °C under shaking. At scheduled times (30 minutes, 1 hour and 2 hours) 8 ml of the supernatant (that was well separated from the solid phase by centrifugation at 5000 rpm for 15 min) was removed for Ca^2+^ and F^−^ quantification by ICP-OES and fluoride ion selective electrode, respectively. After that, samples were rinsed with 8 ml of fresh artificial saliva, and the suspension was again shacked at 37 °C until the next time point.

### *In vitro* remineralization and dentinal tubules occlusion

Five sound human molar teeth extracted for clinical reasons (Oral Surgery Unit, Department of Biomedical, Surgical and Dental Sciences, Milan, Italy) were sectioned horizontally under constant water cooling in order to expose both enamel and dentin, and subsequently four regions were delimited in each sample with a bur-made cross incision. After that, specimens were polished and the enamel and the dentin were etched with H_3_PO_4_ (37 wt% in H_2_O) for 30 seconds, followed by extensive rinsing with ultrapure water.

The Institutional Review Board of the University of Milan approved the use of the teeth, and written informed consent was obtained from each donor. This part of the study was performed according to the principles of the Declaration of Helsinki updated by the World Medical Association in 2013.

*In vitro* experiments were performed treating the sample regions with a 50 wt% slurry of ACP or FACP powders for 24 h, in a 100% humidity atmosphere. Afterwards, the samples were washed for 1 min under running tap water and dried. Samples regions were treated with ACP-4, FACP-h4, ACP-1 and FACP-h1; one part of each specimen was not treated and acted as control.

Specimens were then subjected to critical point drying (Critical-Point Dryer, EMS 850, Hatfield, PA, USA), mounted on stubs with conductive tape, sputter coated (JEOL FFC-1100, Tokyo, Japan), and observed with SEM (Jeol JSM 840 A, Tokyo, Japan) at 15 KV acceleration voltage. Four randomly selected fields at magnifications of 1000x, 2000x, 5000x and 10000x were recorded for each specimen.

## Electronic supplementary material

Supplementary information

## References

[1] Neel EAA (2016). Demineralization–remineralization dynamics in teeth and bone. International Journal of Nanomedicine.

[2] Cochrane NJ, Cai F, Huq NL, Burrow MF, Reynolds EC (2010). New Approaches to Enhanced Remineralization of Tooth Enamel. Journal of Dental Research.

[3] Enax J, Epple M (2018). Synthetic hydroxyapatite as a biomimetic oral care agent. Oral Health and Preventive Dentistry.

[4] Roveri, N. *et al*. Surface Enamel Remineralization: Biomimetic Apatite Nanocrystals and Fluoride Ions Different Effects. *Journal of Nanomaterials* (2009).

[5] Zhang, X., Deng, X. & Wu, Y. In *Nanotechnology in Endodontics: Current and Potential* Clinical *Applications* (ed. Anil Kishen) 173–193 (Springer International Publishing, 2015).

[6] Zhao J, Liu Y, Sun W-b, Zhang H (2011). Amorphous calcium phosphate and its application in dentistry. Chemistry Central Journal.

[7] Ruan Q, Moradian-Oldak J (2015). Amelogenin and enamel biomimetics. Journal of Materials Chemistry B.

[8] Li L (2008). Repair of enamel by using hydroxyapatite nanoparticles as the building blocks. Journal of Materials Chemistry.

[9] Melo MAS, Guedes SFF, Xu HHK, Rodrigues LKA (2013). Nanotechnology-based restorative materials for dental caries management. Trends in Biotechnology.

[10] Betts F, Posner AS (1974). An X-ray radial distribution study of amorphous calcium phosphate. Materials Research Bulletin.

[11] Posner AS, Betts F, Blumenthal NC (1980). Formation and structure of synthetic and bone hydroxyapatites. Progress in Crystal Growth and Characterization.

[12] Boskey AL, Posner AS (1973). Conversion of amorphous calcium phosphate to microcrystalline hydroxyapatite. A pH-dependent, solution-mediated, solid-solid conversion. J. Phys. Chem..

[13] Hannig M, Hannig C (2010). Nanomaterials in preventive dentistry. Nat Nano.

[14] Zhao J, Liu Y, Sun W-b, Yang X (2012). First detection, characterization, and application of amorphous calcium phosphate in dentistry. Journal of Dental Sciences.

[15] Robinson C (2003). Subunit Structures in Hydroxyapatite Crystal Development in Enamel: Implications for Amelogenesis Imperfecta. Connective Tissue Research.

[16] Combes C, Rey C (2010). Amorphous calcium phosphates: Synthesis, properties and uses in biomaterials. Acta Biomater..

[17] Dorozhkin SV (2010). Amorphous calcium (ortho)phosphates. Acta Biomaterialia.

[18] Reynolds EC (1998). Anticariogenic complexes of amorphous calcium phosphate stabilized by casein phosphopeptides: a review. Special Care in Dentistry.

[19] Wang H (2017). Oriented and Ordered Biomimetic Remineralization of the Surface of Demineralized Dental Enamel Using HAP@ACP Nanoparticles Guided by Glycine. Scientific Reports.

[20] Xiao Z (2017). Rapid biomimetic remineralization of the demineralized enamel surface using nano-particles of amorphous calcium phosphate guided by chimaeric peptides. Dental Materials.

[21] Li Y, Weng W (2007). *In vitro* synthesis and characterization of amorphous calcium phosphates with various Ca/P atomic ratios. Journal of Materials Science: Materials in Medicine.

[22] Wu Z (2017). Self-Etch Adhesive as a Carrier for ACP Nanoprecursors to Deliver Biomimetic Remineralization. ACS Applied Materials & Interfaces.

[23] Qi C (2013). Highly Stable Amorphous Calcium Phosphate Porous Nanospheres: Microwave-Assisted Rapid Synthesis Using ATP as Phosphorus Source and Stabilizer, and Their Application in Anticancer Drug Delivery. Chemistry – A European Journal.

[24] Yang X (2011). Influence of magnesium ions and amino acids on the nucleation and growth of hydroxyapatite. CrystEngComm.

[25] Tang Q-L (2010). Porous nanocomposites of PEG-PLA/calcium phosphate: room-temperature synthesis and its application in drug delivery. Dalton Transactions.

[26] Li J (2014). Long-term remineralizing effect of casein phosphopeptide-amorphous calcium phosphate (CPP-ACP) on early caries lesions *in vivo*: A systematic review. Journal of Dentistry.

[27] Rahiotis C, Vougiouklakis G (2007). Effect of a CPP-ACP agent on the demineralization and remineralization of dentine *in vitro*. Journal of Dentistry.

[28] Poggio C, Lombardini M, Vigorelli P, Ceci M (2013). Analysis of dentin/enamel remineralization by a CPP‐ACP paste: AFM and SEM study. Scanning.

[29] Farooq I, Moheet IA, Imran Z, Farooq U (2013). A review of novel dental caries preventive material: Casein phosphopeptide–amorphous calcium phosphate (CPP–ACP) complex. King Saud University Journal of Dental Sciences.

[30] Zero DT (2006). Dentifrices, mouthwashes, and remineralization/caries arrestment strategies. BMC Oral Health.

[31] Lynch R, Navada R, Walia R (2004). Low‐levels of fluoride in plaque and saliva and their effects on the demineralisation and remineralisation of enamel; role of fluoride toothpastes. International Dental journal.

[32] ten Cate JM (2013). Contemporary perspective on the use of fluoride products in caries prevention. Br Dent J.

[33] Lynch R J M, ten Cate J M (2005). The anti-caries efficacy of calcium carbonate-based fluoride toothpastes. International Dental Journal.

[34] Ionescu AC (2017). Streptococcus mutans adherence and biofilm formation on experimental composites containing dicalcium phosphate dihydrate nanoparticles. Journal of Materials Science: Materials in Medicine.

[35] Delgado-López JM (2012). Crystallization of bioinspired citrate-functionalized nanoapatite with tailored carbonate content. Acta Biomater..

[36] Chen Y, Gu W, Pan H, Jiang S, Tang R (2014). Stabilizing amorphous calcium phosphate phase by citrate adsorption. CrystEngComm.

[37] Delgado-López JM (2014). Crystal Size, Morphology, and Growth Mechanism in Bio-Inspired Apatite Nanocrystals. Adv. Funct. Mater..

[38] Chatzipanagis K (2016). Crystallization of citrate-stabilized amorphous calcium phosphate to nanocrystalline apatite: a surface-mediated transformation. CrystEngComm.

[39] Ivanchenko P (2017). On the surface effects of citrates on nano-apatites: evidence of a decreased hydrophilicity. Scientific Reports.

[40] Hu YY, Rawal A, Schmidt-Rohr K (2010). Strongly bound citrate stabilizes the apatite nanocrystals in bone. Proc. Natl. Acad. Sci. USA.

[41] Davies E (2014). Citrate bridges between mineral platelets in bone. Proc. Natl. Acad. Sci. USA.

[42] Zipkin I, Gold RS (1963). The Citrate Content of Teeth. Proc. Soc. Exp. Biol. Med..

[43] Zipkin I, Piez KA (1950). The Citric Acid Content of Human Teeth. J. Dent. Res..

[44] Everett ET (2011). Fluoride’s Effects on the Formation of Teeth and Bones, and the Influence of Genetics. Journal of Dental Research.

[45] Vecstaudza J, Locs J (2017). Novel preparation route of stable amorphous calcium phosphate nanoparticles with high specific surface area. Journal of Alloys and Compounds.

[46] Xu HHK, Moreau JL, Sun L, Chow LC (2011). Nanocomposite containing amorphous calcium phosphate nanoparticles for caries inhibition. Dental Materials.

[47] Iafisco M (2015). The growth mechanism of apatite nanocrystals assisted by citrate: relevance to bone biomineralization. CrystEngComm.

[48] Chen, Y., Gu, W., Pan, H., Jiang, S. & Tang, R. Stabilizing amorphous calcium phosphate phase by citrate adsorption. *CrystEngComm*, (2013).

[49] Hartles RL (1964). Citrate in Mineralized Tissues. Adv. Oral Biol..

[50] Dorozhkin SV (2016). Calcium orthophosphates (CaPO4): occurrence and properties. Progress in Biomaterials.

[51] Chen J (2015). Effects of fluorine on the structure of fluorohydroxyapatite: a study by XRD, solid-state NMR and Raman spectroscopy. Journal of Materials Chemistry B.

[52] Arends J (1992). Rate and mechanism of enamel demineralization *in situ*. Caries research.

[53] Øgaard B, Rølla G, Arends J (1988). Orthodontic appliances and enamel demineralization: Part 1. Lesion development. American Journal of Orthodontics and Dentofacial Orthopedics.

[54] Angelini E, Bianco P, Mascellani S, Zucchi F (1993). Low-noble metal alloys: *in vitro* corrosion evaluation. Journal of Materials Science: Materials in Medicine.

[55] Duffó G, Castillo EQ (2004). Development of an artificial saliva solution for studying the corrosion behavior of dental alloys. Corrosion.

