# Supplementary material for: Fluoride-doped amorphous calcium phosphate nanoparticles as a promising biomimetic material for dental remineralization
Source: Sci Rep. 2018 Nov 19;8:17016. doi: 10.1038/s41598-018-35258-x (PMC6242929; doi:10.1038/s41598-018-35258-x)
Supplement: Supplementary file 1 — Supplementary information [file 41598_2018_35258_MOESM1_ESM.pdf]

Supplementary Information for

Fluoride-doped amorphous calcium phosphate nanoparticles as a promising biomimetic material for dental remineralization

Michele Iafisco,[*a] Lorenzo Degli Esposti,[a,b] Gloria Belén Ramirez Rodríguez,[c] Francesca Carella,[a] Jaime Gomez Morales,[d] Andrei Ionescu,[e] Eugenio Brambilla,[e] Anna Tampieri[a] and José Manuel Delgado Lopez[*c]

a. Institute of Science and Technology for Ceramics (ISTEC), National Research Council (CNR), Via Granarolo 64, 48018 Faenza (Italy). E-mail: michele.iafisco@istec.cnr.it

b. Department of Chemistry, Life Sciences and Environmental Sustainability, University of Parma, Parco Area delle Scienze 17/a, 43124 Parma (Italy).

c. Department of Inorganic Chemistry, University of Granada, Av. Fuente Nueva, s/n, 18071, Granada, Spain. E-mail: jmdl@ugr.es

d. Laboratorio de Estudios Cristalográficos, Instituto Andaluz de Ciencias de la Tierra, IACT (CSIC-UGR), Av. Las Palmeras 4, 18100 Armilla (Spain).

e. Oral microbiology Laboratory, Galeazzi Orthopedic Institute, Department of Biomedical, Surgical and Dental sciences, University of Milan, Via Pascal, 36, 20133 Milan (Italy)

Sample	Cit/Ca ratio	NaF (mM)	$CaCl_2$ (mM)	$Na_3(Cit)$ (mM)	Na_2HPO_4 (mM)	Na_2CO_3 (mM)
ACP-4	4	-	100	400	120	200
FACP-l4	4	5	100	400	120	200
FACP-h4	4	50	100	400	120	200
ACP-2	2	-	100	200	120	200
FACP-h2	2	50	100	200	120	200
ACP-1	1	-	100	100	120	200
FACP-h1	1	50	100	100	120	200

Table S1. Codes, Cit/Ca molar ratio and concentrations of reactants used for the preparation of the samples.

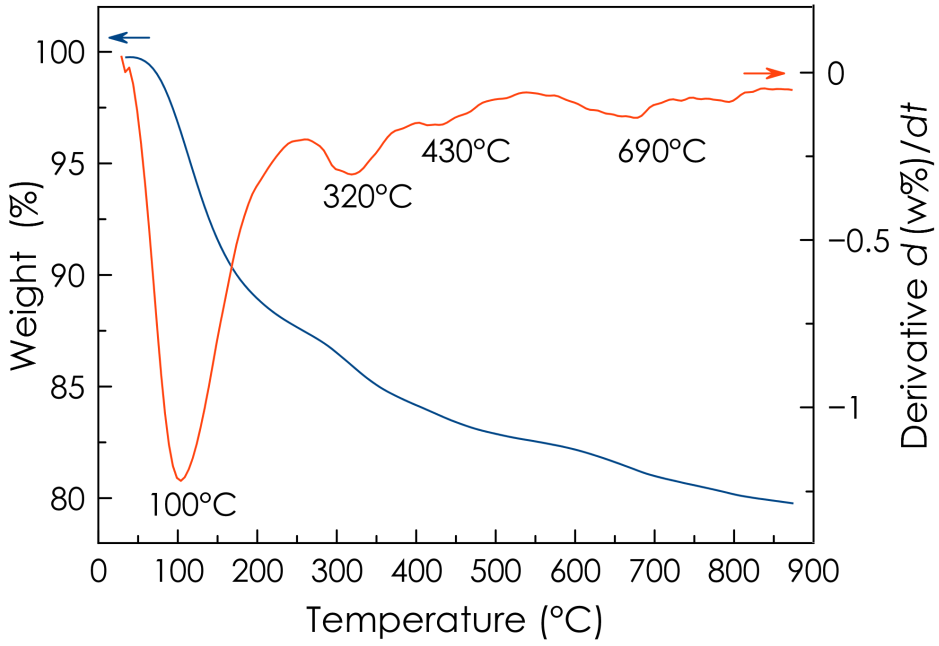

Figure S2. TGA curve and first derivative for ACP-4 sample.

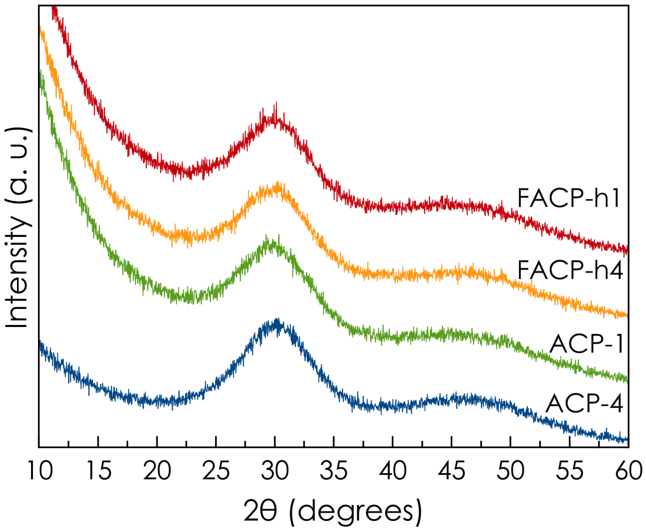

Figure S3. PXRD pattern of ACP-4, ACP-1, FACP-h4, FACP-h1, one year after synthesis, stored at room temperature.

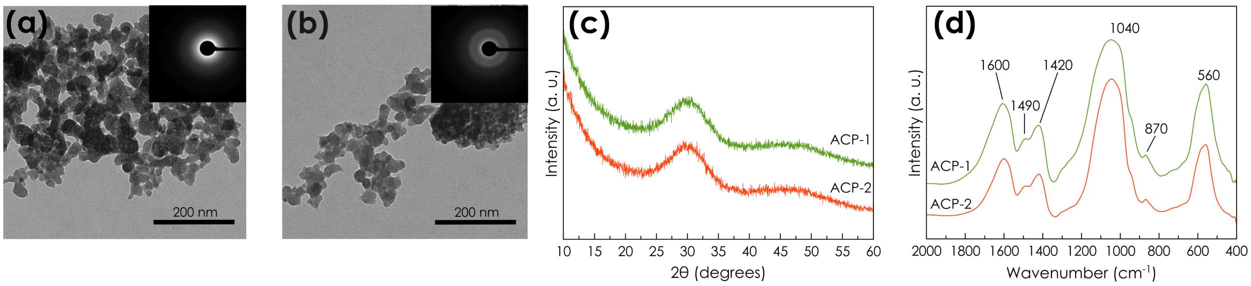

Figure S4. TEM micrograph of (a) ACP-2 and (b) ACP-1 nanoparticles (insets: SAED patterns); (c) XRD patterns and (d) FTIR spectra of ACP-2 (red) and ACP-1 (green) nanoparticles.

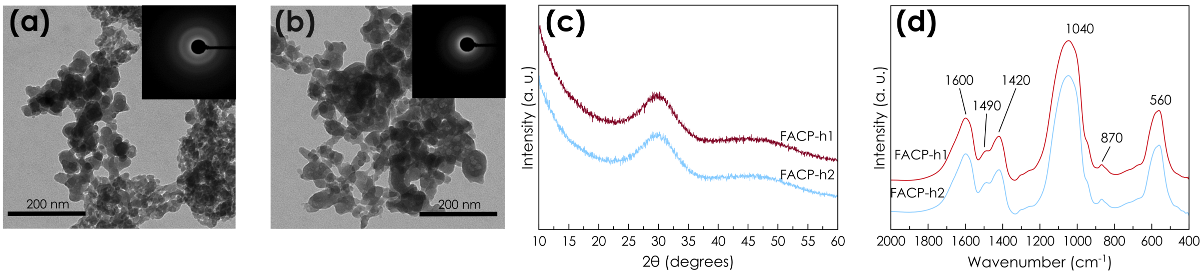

Figure S5. TEM micrograph of (a) FACP-h2 and (b) FACP-h1 nanoparticles (insets: SAED patterns); (c) XRD patterns and (d) FTIR spectra of FACP-h2 (blue) and FACP-h1 (magenta) nanoparticles.

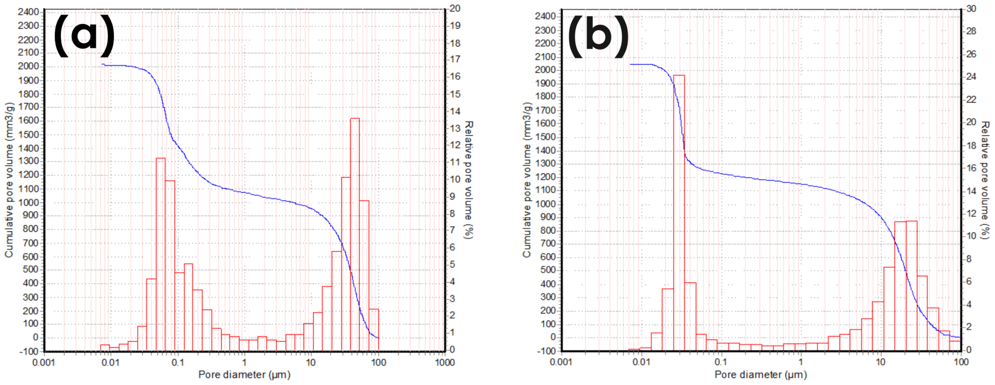

Figure S6. Pore size distribution histogram for (a) ACP-4 and (b) ACP-1.

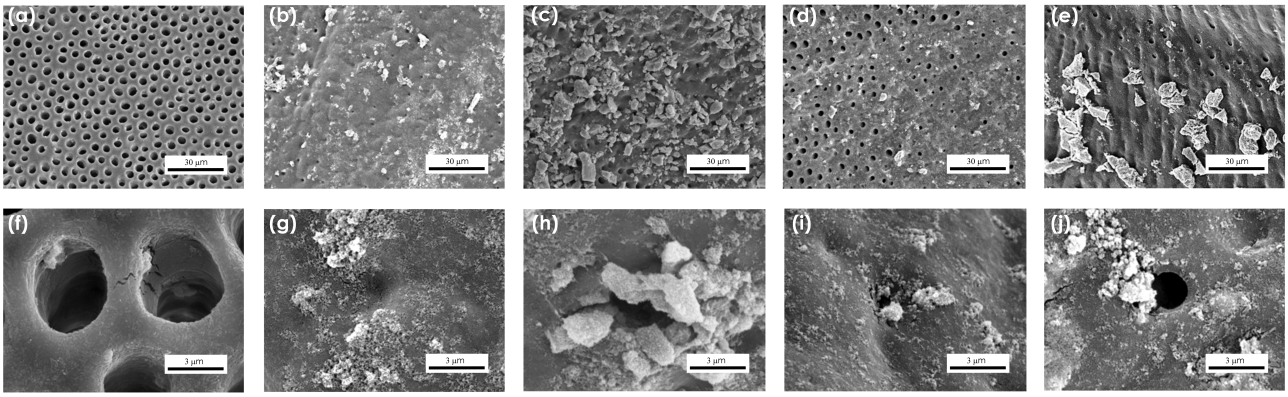

Figure S7. SEM micrographs of (a, f) demineralized dentin and demineralized dentin treated with (b, g) ACP-4, (c, h) FACP-h4, (d, i) ACP-1, and (e, j) FACP-h1 at two different magnifications.

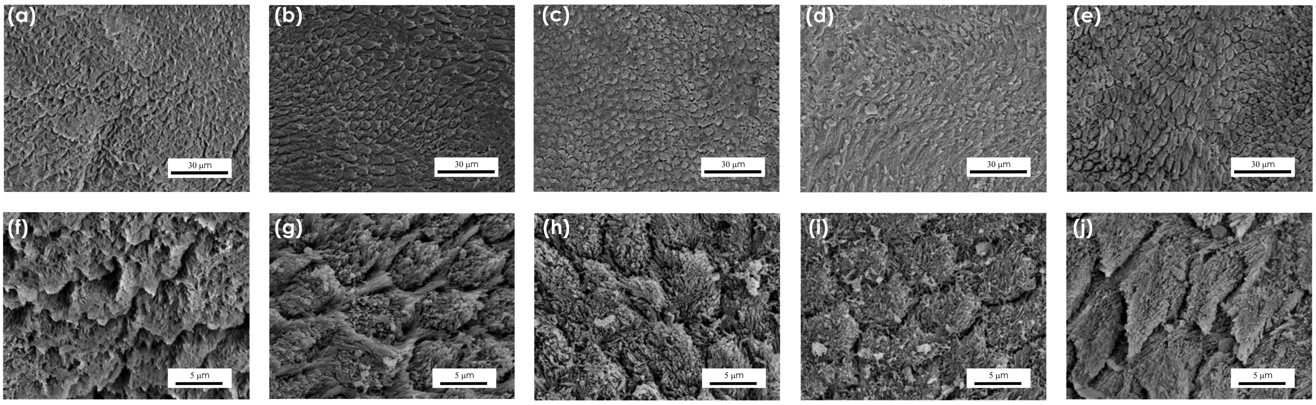

Figure S8. SEM micrographs of (a, f) demineralized enamel and demineralized enamel treated with (b, g) ACP-4, (c, h) FACP-h4, (d, i) ACP-1, and (e, j) FACP-h1 at two different magnifications.
